# Editorial: Fatigue assessment in sport

**DOI:** 10.3389/fspor.2023.1131297

**Published:** 2023-02-13

**Authors:** Valentina Agostini, Karla De Jesus, Pietro Picerno

**Affiliations:** ^1^ PolitoBIOMedLab, Department of Electronics and Telecommunications, Polytechnic University of Turin, Turin, Piedmont, Italy; ^2^ Human Performance Studies Laboratory, Faculty of Physical Education and Physiotherapy, Federal University of Amazonas, Manaus, Amazonas, Brazil; ^3^ SMART Engineering Solutions & Technologies, University of eCampus, Novedrate (CO), Italy

**Keywords:** muscle fatigue, excercise-induced fatigue, running, swimming, electrical stimulation

Editorial on the Research TopicFatigue assessment in sport

To avoid excessive exercise-induced load and overtraining, or to facilitate a proper recovery after a sport-related injury, it is essential to adequately monitor exercise intensity and manage fatigue (1). In this perspective, sport science and technology continuously propose advanced techniques to tackle the challenges of coaches and athletes. It is crucial to rely on objective measurements of fatigue in order to tailor training sessions and recovery programs to the specific needs of each individual professional or amateur athlete (2). However, fatigue is a multi-faced phenomenon requiring innovative and integrated approaches, including the evaluation of the manifestations of muscle fatigue in surface electromyograms recorded during an exercise, and the study of central and peripheral components of fatigue. This Research Topic aims to provide solutions beyond the state of the art for accurately and effectively monitoring fatigue during sporting performance and exercise training. Indeed, properly managing fatigue and fatigue-related symptoms can be a key factor for limiting athletes' musculoskeletal problems or altered proprioception that can cause traumas. This Research Topic was not limited to contributions related to elite athletes and elite para-athletes but analyzed also studies related to amateur practitioners of physical activities for fitness and recreational purposes. The present research topic collected a total of 7 research articles covering different sports, themes and issues. Within these articles, we identified three areas of research with respect to the impact of fatigue assessment in sport: fatigue in running (Prigent et al.; Gao et al.; Gao et al.), fatigue in swimming (Puce et al.; Puce et al.), techniques to manage fatigue (Abitante et al.; Bouchiba et al.).

## Fatigue in running

Prigent and coll. analyzed the evolution of biomechanical- physiological-, and psychological-related factor of muscular fatigue during a half marathon in 13 amateur runners equipped with a foot-mounted inertial sensor, a chest-mounted hart rate monitor with GPS, and a smartphone for recording, every 10 min of race, a perceived rating of fatigue (Prigent et al.). The authors showed that the perceived fatigue relates with the actual biomechanical and physiological demands in faster runners, concluding that their performance can be associated to their ability of better judging and managing their physiological limits. The work authored by Gao and coll. addressed the issue of the influence of gait symmetry in running-related injury and, in particular, it aimed at assessing the effects of running fatigue on the symmetry of lower limb biomechanical parameters in eighteen male amateur runners wearing the same model of shoes (Gao et al.). Lower limb joint kinematics and kinetics were collected during a 10-m overground running at a comfortable speed before and after a fatiguing protocol. Results showed that running fatigue resulted in an increased asymmetry of load on the hip, knee and ankle joints of the lower extremities while not necessarily deteriorating the symmetry of all biomechanical parameters especially the joint angles in the horizontal plane. Another study by Gao and coll. investigated the changes in knee joint symmetry during a Counter-Movement Jump (CMJ) task, before and after running-induced fatigue, in recreational runners (Gao et al.). After the prolonged running protocol, a significant increase in the knee joint angle asymmetry was found in the horizontal plane, during the push-off and landing stage. These increases in asymmetry are mainly caused by excessive external rotation of the dominant knee joint. Future studies are needed to confirm the association between knee joints asymmetry and fatigue, and to investigate the possible relationship with jump-related injuries.

## Fatigue in swimming

A swimming tactic (i.e., drafting), in which an athlete swims in the wave of another athlete, was analyzed in the work authored by Puce and coll. in terms of muscle fatigue and swimming efficiency (Puce et al.). “Behind Drafting” was found to be the best swimming configuration for the drafter. Considering Para swimmers, another study by Puce and coll. highlighted that only subject with spastic dystonia (but not those with spasticity) the hypertonia worsens after fatigue, and pain develops (Puce et al.). Therefore, the technical staff and medical classifiers should be aware of this specificity, because intense and prolonged motor activity could negatively affect competition performance in athletes with spastic dystonia, creating a situation of unfairness between Para athletes belonging to the same sports class.

## Techniques to manage fatigue

The purpose of the research paper authored by Abitante and coll. was to explore the effect of exercise training typology (endurance vs. explosive) on muscle fatigability during repetitive contractions induced by Neuromuscular Electrical Stimulation (NMES) (Abitante et al.). Thirty-four subjects were placed in a leg extension rig and received 1 s of NMES every 3 s and for 20 min to their vastus medialis muscle, resulting in isometric leg extensions against a fixed resistance. The endurance group fatigued significantly less than both the explosive and control cohorts, with no difference observed between the explosive and the controls, suggesting that when designing a NMES regimen for athletic training, the training of the specific athlete should be taken into account. Among the techniques to manage fatigue, the study by Bouchiba and coll. highlighted that Cold Water Immersion (CWI) might be an interesting strategy to recover from both central and peripheral fatigue (Bouchiba et al.).

The assessment of muscle fatigue in sport is crucial for the high impact that can have in the coaching process of both professional and amateur athletes. The present collection of research articles have added further insights to fatigue in running, swimming, as well as in techniques to manage fatigue such as NMES and CWI. The continuous technological advances in the field of measurement techniques allow researchers to collect biomechanical- and physiological-related data in the wild and for long period of time (3). It is, hence, desirable that the research interest towards the assessment of muscle fatigue in sport may grow in the next future as new frontiers in data collection can be explored.

## References

[1] Halson SL. Monitoring training load to understand fatigue in athletes. Sports Med. (2014) 44(Suppl 2):139–47. 10.1007/s40279-014-0253-z PMC4213373 25200666

[2] Bestwick-Stevenson T Toone R Neupert E Edwards K Kluzek S. Assessment of fatigue and recovery in sport: narrative review. Int J Sports Med. (2022) 43(14):1151–62. 10.1055/a-1834-7177 35468639

[3] Dorschky E Camomilla V Davis J Federolf P Reenalda J Koelewijn AD. Perspective on “in the wild” movement analysis using machine learning. Hum Mov Sci. (2022) 87:103042. 10.1016/j.humov.2022.103042 36493569

